# Crash Frequency Modeling Using Real-Time Environmental and Traffic Data and Unbalanced Panel Data Models

**DOI:** 10.3390/ijerph13060609

**Published:** 2016-06-18

**Authors:** Feng Chen, Suren Chen, Xiaoxiang Ma

**Affiliations:** 1 Key Laboratory of Road & Traffic Engineering of the Ministry of Education, Tongji University, 4800 Cao’an Road, Shanghai 201804, China; fengchen@tongji.edu.cn; 2 Department of Civil & Environmental Engineering, Colorado State University, Fort Collins, CO 80523, USA; xiaoxiang.ma@colostate.edu

**Keywords:** hourly crash frequency, real-time driving environment, unbalanced panel data, zero-inflated negative binomial, refined temporal scale

## Abstract

Traffic and environmental conditions (e.g., weather conditions), which frequently change with time, have a significant impact on crash occurrence. Traditional crash frequency models with large temporal scales and aggregated variables are not sufficient to capture the time-varying nature of driving environmental factors, causing significant loss of critical information on crash frequency modeling. This paper aims at developing crash frequency models with refined temporal scales for complex driving environments, with such an effort providing more detailed and accurate crash risk information which can allow for more effective and proactive traffic management and law enforcement intervention. Zero-inflated, negative binomial (ZINB) models with site-specific random effects are developed with unbalanced panel data to analyze hourly crash frequency on highway segments. The real-time driving environment information, including traffic, weather and road surface condition data, sourced primarily from the Road Weather Information System, is incorporated into the models along with site-specific road characteristics. The estimation results of unbalanced panel data ZINB models suggest there are a number of factors influencing crash frequency, including time-varying factors (e.g., visibility and hourly traffic volume) and site-varying factors (e.g., speed limit). The study confirms the unique significance of the real-time weather, road surface condition and traffic data to crash frequency modeling.

## 1. Introduction

Despite all the efforts during the past decades, traffic crashes are still the primary threat on highways in most countries. A better understanding of the critical contributing factors and the ability to predict the crash risk has become the key to various prevention efforts, such as advanced traffic management, proactive law enforcement, and injury mitigation. Traditionally, most crash frequency models used aggregated information with relatively large time scales (e.g., yearly), rather than detailed, time-varying data in smaller time scales (e.g., hourly, daily, or weekly). Real time traffic and environmental conditions (e.g., weather conditions) have significant impact on crash occurrence, therefore the large scales and aggregated variables may not be sufficient for some complex or adverse driving conditions, such as inclement weather and/or complex terrains.

As a result of adopting larger time scales, some important information of critical driving environmental variables over time (e.g., weather or traffic data) is often lost [1]. Therefore, the crash frequency models developed with aggregated data can only provide the results based on average or cumulative data over longer time periods, which may lose potentially important explanatory information and also introduce error due to unobserved heterogeneity [2]. In addition to possible error, some real-time driving environmental variables may not be found significant until more refined data and temporal scales are used in the model. This is especially critical for locations where some explanatory variables experience considerable variations temporally (e.g., inclement weather, rush hours).

Although it seems obvious that crash frequency models with more refined temporal scales are more desirable, to develop appropriate models with detailed time-varying and spatial-varying data is not straightforward. By using more refined data in temporal domain, the same road segment may generate multiple observations, which will be correlated over time by sharing unobserved effects [1]. The temporal correlations, if they exist, pose methodological challenges on rationally predicting crash frequency. This is likely another reason that people often attempt to use more aggregated data to develop crash frequency models, although some useful information of the explanatory variables are inevitably lost.

In recent years, with the popularity of ITS applications around the world, rich data source, including continuously monitoring real-time data, becomes more readily available on many major highways. With the detailed monitoring data, some attempts have been made to develop crash frequency models with more refined scales, which primarily focus on real-time relative crash risk or likelihood. There are, however, very few studies on the modeling of crash frequency in refined scales and more details can be found in the following literature review subsections. The present study reports the recent efforts on developing crash frequency models in refined temporal scales using disaggregated and unbalanced panel-data structure. Zero-inflated negative binomial models with random effects are developed using panel data in the present study to deal with temporal correlation as well as dominating zero observations. The inherent correlations of observations with a comprehensive coverage of all major contributing factors, including real-time environmental conditions, will also be appropriately considered. Interstate highway I-25 in Colorado will be studied to demonstrate the methodology and provide some interesting findings.

### 1.1. Real-Time Crash Risk Models in Refined Temporal Scales

As discussed earlier, the adoption of aggregated data may cause some important detailed information being lost in the model [1]. In recent years, in addition to crash frequency and crash rate models, many studies have emerged primarily developing real-time crash frequency models which estimate the likelihood of crash occurrence using short-term traffic and environmental conditions [3,4,5,6,7,8,9,10,11,12,13,14,15,16].

In these studies, historical crash data has been typically linked with real-time traffic and environmental data. In most of these studies, rather than direct crash frequency modeling, the relative crash probability was often predicted and compared with the crash probability under conditions without crashes [14]. For example, the matched case–control design has been frequently utilized in these crash probability studies [12,14,16,17], in which several (e.g., four) non-crash cases were matched for each specific crash case. Some other methods have also been adopted to develop the crash probability models, including neural network [18] and Bayesian network [10]. In these studies, the data structure was based on case-control of crash records instead of the data with both spatial and time varying information for road segments. Roshandel *et al.* [19] and Theofilatos [20] reviewed the papers about real-time freeway crash modeling to provide a summary impact of traffic and weather characteristics on crash occurrence. Therefore, the present study is different from these existing studies by developing a direct crash frequency model for road segments rather than relative crash probability models.

### 1.2. Crash Frequency Models: Panel Data Application and Zero-Inflated Consideration

By directly quantifying crash counts, crash frequency modelling is an important tool to study crash risks on highways. A significant number of count models have been developed to predict crash frequency during the past several decades. The Poisson model is a popular starting point among various count models, with the negative binomial (NB) model being an extension of the Poisson model to handle crash data with over-dispersion issues. In social and behavioral science, panel data models have been extensively used for data with both spatial and time varying information while still taking account of the heterogeneity of the individuals. Because road crash data also has cross-sectional and time-serial nature, panel data count models, such as fixed effects or random effects Poisson models and negative binomial models, have been adopted for crash frequency analysis in recent years. For instance, Noland [21] and Noland and Oh [22] used the fixed negative binomial models to study the impacts of roadway infrastructure improvements on fatal and injured traffic crash frequencies based on the aggregate state-wide and county-level data. The fixed effects Poisson or negative binomial models, which are conditioned on the total number of observed crashes, do not allow for site-specific or time-specific variations.

To deal with such a limitation, Shankar *et al.* [23] first developed a random effects negative binomial model to investigate the impacts of geometric and traffic factors on median crossover crash frequency. In addition to random effects negative binomial models, some other random effect or random parameter crash frequency models have also been explored [24,25,26,27,28,29,30]. Anastasopoulos and Mannering [27] developed a random parameter negative binomial model to predict annual crash frequency using 9-year data. Aguero-Valverde [29] compared random effect Poisson-gamma and Poisson-lognormal models and traditional Poisson-gamma and Poisson-lognormal models when before- and after- analyses of road safety countermeasures were carried out. In addition to panel data modeling, it is worth mentioning that the negative multinomial model can also be used to investigate temporal and cross-sectional variations simultaneously. For example, the negative multinomial model using a multi-year panel of cross-sectional roadway data was developed to predict the number of median crossover crashes by Ulfarsson and Shankar [31]. In the study conducted by Caliendo *et al.* [32], Poisson, negative binomial and negative multinomial regressions were compared in terms of predicting crash frequency of multi-lane roads. The main focuses of these panel crash frequency models were to deal with correlated data caused by yearly repeated observations (multi-year crash frequency) reflecting long-term effects of contributing factors. For example, when traffic flow and weather information were being considered, existing crash frequency model applications usually utilized long-term average data, such as annual average daily traffic volume and/or annual days with rainfall in a year [33].

One challenge associated with crash frequency modelling is excess zero crash observations, especially when the sample scale is reduced. As a result, excessive zeroes in the records need to be taken care of if a refined-scale model with panel data is to be developed. As an extension of standard Poisson and negative binomial regression, Zero-inflated Poisson (ZIP) and Zero-inflated negative binomial (ZINB) models have attracted considerable attention [23,34,35,36]. Although also facing some criticism [37,38], these models are found to provide a statistically superior fit to the data in some recent applications [39,40]. Some efforts have also been made using random effect or random parameter zero-inflated models to predict annual crash frequency. Huang and Chin [41] tried to use a random effects zero-inflated Poisson regression to study the crash frequency using 8-year crash data in Singapore in a yearly temporal scale. Dong *et al.* [30] adopted a multivariate random-parameter, zero-inflated, negative binomial regression model to estimate annual crash frequencies at intersections using 5-year data. While crashes are extremely rare over the considered time period (e.g., one day or one hour), the zero-crash state may be presented as a reasonable theoretical and empirical construct for the description of dominating virtually safe states on some roadway segments [42]. Recently, generalized ordered response models which subsume standard count models as subcases and provide more flexibility than zero-inflated models were developed [43]. For example, Castro *et al.* [43] proposed an equivalent latent variable-based generalized ordered response framework to study crash frequency at urban intersections, which can also handle excess zeros in correlated count data. But so far, studies which focus on developing crash frequency models with refined temporal scales are still rare. This paper intends to contribute to the literature by developing refined scale crash frequency models while addressing zero-inflation and serial correlations simultaneously. Although the present method is more time consuming to gather all non-accident cases, it can avoid some key information loss including both spatial and time varying information for road segments.

## 2. Data Description

In preparation for this study, we first establish a comprehensive crash database containing information on crash record, road design, real-time traffic flow, weather conditions and road surface conditions. The database includes hourly distributions of crash, traffic, weather and road surface data for each roadway segment (in average 1-mile length) for both driving directions of one portion of interstate I-25 in Colorado, with the total length of 55.93 miles. A relational database is assembled with information from four sets of data in this study: (1) one year of crash database (from January 2010 to January 2011) provided by the Colorado State Patrol (CSP); (2) road segment geometric characteristic data provided by the Colorado Department of Transportation (CDOT); (3) real-time weather and road surface condition data recorded by five weather stations along the I-25 roadway segment; and (4) real-time traffic data detected by forty-three traffic flow monitoring stations along this segment. The combination of these data sets provides a very rich source of information that allows us to comprehensively study almost all the possible factors influencing crash frequency in refined scales. It should be noted that the real-time weather, road surface condition and traffic data in this study is primarily from the Road Weather Information System (RWIS), which is available on many major highways across the United States. The dependency on the data from RWIS offers significant advantage over very rare or inaccessible data source in terms of conveniently transferring the proposed technology to other highways without additional investments on data collection facilities.

The I-25 corridor in Colorado being studied is between the City of Castle Pines and the City of Northglenn which includes segments across the City and the County of Denver. The 28.55-mile north bound portion of I-25, starting at mile marker (MM) 188.49 and ending at MM 221.03, is split into 29 segments. Similarly, the 27.38-mile south bound potion of I-25, starting at MM 188.49 and ending at MM 219.86, is split into 28 segments with an average length of each segment being around 1 mile. The segments are split in a homogeneous pattern based on changes of geometric features, including curve, longitudinal grade, speed limit *etc.* according to the CDOT Roadway Characteristics Inventory (RCI) and traffic station assignment. If a distinct variance of road design within one road segment exists (e.g., variance of lane width, number of lanes, speed limit, shoulder type, median type), the road segment will be re-segmented based on different geometric designs.

The corresponding traffic flow and environmental data of each roadway segment is also used in the analysis. Information about temperature, visibility, humidity, wind and precipitation, and road surface conditions is provided by the RWIS. The RWIS stations report frequent readings as the weather conditions change within a short time period. For example, visibility in general can be described as the maximum distance that an object can be clearly perceived against the background sky. We choose the lowest clear distance in miles that drivers can see in any hour as an hourly measure of visibility. In this study, the detailed precipitation and road surface condition data for each geographical location and time period is also obtained. Road surface condition types defined in the CDOT database include Dry, Wet, Trace Moisture, Chemically Wet (moisture mixed with anti-icer), Ice Warning, Ice Watch and so on. Each segment of the study has been assigned to the nearest weather station according to the mile marker. The weather stations report the weather and surface conditions with 20-min intervals in average and the raw data is combined into the data with 1-h interval. The hourly road surface condition is defined with the dominant road surface condition type of that particular hour period. For example, if the weather station recorded two times of wet road surface and one time of dry road surface in one given hour, the hourly road surface condition will be determined as wet road surface. Therefore, for each segment and each hour, the hourly average weather record closest to the road segment has been extracted and used as the hourly environmental condition of that particular road segment.

In the proposed model, we derive directional hourly traffic volumes for all road segments from 43 traffic stations. There are 22 and 21 traffic stations located on the north and south bounds respectively which provide speed, volume and occupancy information. The sensors record 2-min aggregation of speed, volume and occupancy, and the hourly average speed, volume and occupancy for each segment are calculated from this data. Real-time traffic speed influences crash probability, but is also partially controlled by the speed limit of each road segment (the upper limit of the real-time speed is the legal speed limit in the CDOT database). Thus we choose both the speed limit and the difference between the speed limit and the current traffic speed (*i.e.*, speed limit minus traffic speed) to facilitate following analysis.

Temporal dummy variables including night indicator, sunrise indicator and sunset indicator are calculated based on the 2010 Colorado Sunrise Sunset Calendar for each hour. Other temporal variables are in terms of month, day of the week, and hour of the day representing the influences of temporal distribution on crash frequency. One of the traffic characteristics, truck percentage, adopts the peak time truck percentage value between 6–8 am and 4–6 pm, with the off-peak truck percentage falling in all remaining hours of a day.

Note that the real-time data is not recorded in a perfectly continuous manner due to possible malfunction of the data loggers or disruptions. For example, sometimes some weather stations may lose power and engineers may not be able to find and fix the problem promptly. As a result, some empty “windows” may exist in the weather, road surface and traffic data records. The sample thus comprises a total of 328,529 observations (one observation for one road segment in an hour, totaling for 57 road segments and for 365 × 24 h) after deleting those observations without real-time traffic or environmental data. Table 1 summarizes the characteristics of the 328,529 observations, which are statistically significant variables (*p*-value < 0.1) in the final models. For example, November indicator is included in Table 1 because other month indicators are not found statistically significant. The crashes have been assigned to each segment according to the mile marker (MM). A total of 1352 crashes occurred at the corresponding road segments during the one-year period are considered in the analysis. A total of 99.6% of observations are zeroes (for one road segment in an hour). The data exhibit over-dispersed as the mean and std. dev. of crash frequency is equal to 0.004 and 0.066, respectively (Table 1). Only statistically significant variables (*p*-value < 0.1) are included in the final models to capture the crash characteristics on I-25 in Colorado.

## 3. Methods

To relax the over-dispersion constraint imposed by the Poisson model, a negative binomial distribution is commonly used [23,34,44,45].

The negative binomial distribution is shown as:

(1) $$P(n_{it})=\frac{\Gamma ((1/\alpha )+n_{it})}{\Gamma (1/\alpha )\Gamma (n_{it}+1)}{\left(\frac{1/\alpha }{(1/\alpha )+\lambda_{it}}\right)}^{1/\alpha }{\left(\frac{\lambda_{it}}{(1/\alpha )+\lambda_{it}}\right)}^{n_{it}}$$

where Γ is the factorial function, $n_{it}$ is the number of crashes on roadway segment *i* during period *t*, $P(n_{it})$ is the probability of $n_{it}$ crashes occurring on this observation, α is an additional estimable coefficient and $\lambda_{it}$ is the Poisson parameter which equals the expected value of $n_{it}$($E(n_{it})$):

(2) $$\lambda_{it}=exp(\beta_{NB}X_{NB}^{it})$$

where $\beta_{NB}$ is the vector of unknown regression coefficients, and $X_{NB}^{it}$ is the vector of covariates determining crash frequency on roadway segment *i* in time period *t*, such as the roadway segment geometric characteristics and environmental characteristics.

Zero-inflated negative binomial (ZINB) regression models have been developed to address the possibility of zero-inflated crash state. One process has the roadway segment in a non-negative count state for crash frequency (*i.e.*, a normal count process for crash frequency that has a frequency outcome determined by negative binomial distribution). Another process is the zero-crash state where the roadway segment is virtually safe during a specific time period, which may be qualitatively different from Poisson or negative binomial distributed crash frequency counts.

ZINB assumes that the events $n_{it}$ (roadway segment *i* in time period *t*) are independent, and:

(3) $$P[n_{it}=0]=q_{it}+[1-q_{it}]R_{it}(0)$$

(4) $$P[n_{it}=j>0]=[1-q_{it}]R_{it}(j)$$

where:

(5) $$R_{it}(y)=\frac{\Gamma ((1/\alpha )+y)}{\Gamma (1/\alpha )\Gamma (y+1)}{\left(\frac{1/\alpha }{(1/\alpha )+\lambda_{it}}\right)}^{1/\alpha }{\left(\frac{\lambda_{it}}{(1/\alpha )+\lambda_{it}}\right)}^{y}$$

where the definitions of the parameters are the same as the basic negative binomial models, except that the general formulation of *q_it_* is defined as:

(6) $$q_{it}=\exp(\beta_{z}X_{z}^{it})/(1+\exp(\beta_{z}X_{z}^{it}))$$

where $\beta_{z}$ is the estimated coefficient vector in zero-crash state and $X_{z}^{it}\;$ are the vectors of variables of roadway segment *i* during period *t* in zero-crash state.

In the standard Poisson, NB and ZINB models, it is assumed that observations are independent and such an assumption is possibly violated in repeated measures such as crash counts at the same specific site during different time periods. There is almost certain correlation among repeated observations at a specific site due to some unobserved crash-induced factors. Hence, it is necessary to consider the site-specific effects in the ZINB model, especially when repeated measures inevitably occur for disaggregated data considering time-varying effects. ZINB with site-specific random effects can be expressed in the following.

We denote the total number of observations as *N*:

(7) $$N=\sum_{i=1}^{I}t_{i}$$

where *i = 1*, ... , *I,* and *t_i_* is the number of repeated observations in site *i* (site-specific panel data structure), *I* is the total number of different sites. For balanced panel data, *t_i_* is the same for all sites. Because the real-time weather, road surface and traffic data was not recorded in a perfectly continuous manner, *t_i_* is not all the same and thus the panel data structure here was actually unbalanced.

The zero-inflated Negative Binomial model with site-specific random effects is shown as,

(8) $$n_{it}=0$$

with the probability of:

(9) $$q_{it}+(1-q_{it}){\left[\frac{1/\alpha }{(1/\alpha )+\lambda_{it}}\right]}^{1/\alpha }$$

(10) $$n_{it}=y;(y=1,2,\;\ldots )$$

with the probability of:

(11) $$(1-q_{it})\left[\frac{\Gamma ((1/\alpha +y)u_{it}^{1/\alpha }{\left(1-u_{it}\right)}^{y}}{\Gamma (1/\alpha )y!}\right]$$

where:

(12) $$u_{it}=(1/\alpha )[(1/\alpha )+\lambda_{it}]$$

(13) $$\lambda_{it}=exp(\beta_{NB}X_{NB}^{it}+\sigma_{i})$$

(14) $$\text{logit}(q_{it})=\ln(\frac{q_{it}}{1-q_{it}})=\beta_{z}X_{z}^{it}+\psi_{i}$$

$\text{The definitions of other parameters are the same as }\mathrm{previous}\text{ equations}.\;\sigma_{i}$ and $\psi_{i}$ are the site-specific random effects for the two states with independent normal distributions, *i.e.*, $\sigma_{i}\sim N\left(0,\varphi_{\sigma_{i}}^{2}\right)$ and $\psi_{i}\sim N\left(0,\varphi_{\psi_{i}}^{2}\right)$ ($\varphi_{\sigma_{i}}$ and $\varphi_{\psi_{i}}$ are the standard deviations of $\sigma_{i}$ and $\psi_{i}$).

Although it is obvious that there are dominating zero crash observations in the refined panel data, questions still remain about whether zero-inflated crash frequency models are truly statistically more appropriate than traditional counterparts. To test the appropriateness of adopting a zero-inflated model, Vuong [46] proposed a t-statistic-based test where the statistic is determined through firstly computing *m_it_*:

(15) $$m_{it}=\ln\left(\frac{f_{1}(y_{it}|X_{it})}{f_{2}(y_{it}|X_{it})}\right),$$

where $f_{1}\left(y_{it}|X_{it}\right)$ is the probability density function of the zero-inflated negative binomial model and $f_{2}\left(y_{it}|X_{it}\right)$ is the probability density function of the parent negative binomial distribution.

Vuong’s statistic is computed as [23,47]:

(16) $$V=\frac{\bar{m}\sqrt{N}}{S_{m}}$$

where $\bar{m}$ and $S_{m}$ are the mean and the standard deviation of m, respectively. *N* is the sample size. The Vuong’s statistic *V* as defined in Equation (16) is asymptotically and standard normally distributed, so if the absolute value of *V* is less than 1.96 (the 95% confidence level for the *t*-test), the test favors the normal negative binomial. Similarly, the zero-inflated regression model is preferred if the absolute value of *V* is greater than 1.96 [47]. To carry out the test, both the parent and zero-inflated models need to be estimated and tested using *t*-statistic. Statistical software SAS version 9.3 (SAS Institute Inc., Cary, NC, USA) is used for the modeling.

## 4. Results

The model results for the panel data zero-inflated negative binomial estimations with site-specific random effects are presented in Table 2. The estimation results of unbalanced panel data zero-inflated negative binomial models suggest that there are many factors influencing the crash frequency on I-25 including time-varying factors (e.g., visibility and hourly traffic volume) and site-varying factors (e.g., speed limit and number of lanes). A number of factors, which significantly influence the frequency of crashes, are identified, including those of environmental, traffic, temporal, and road characteristics.

The random effects parameters are significant at 99.9% level, which confirms the appropriateness of adopting random effect specification (*t*-statistic for *σ_i_* is 7.54). The over-dispersion parameter *α* is statistically significant (*t*-statistics of 3.57), which implies the negative binomial model is indeed preferred over the Poisson model. The selection of zero-inflated model is endorsed by the Vuong’s test results for zero-inflation (V = 4.48 for model with site-specific random effects). Therefore, random effect zero-inflated negative binomial model is confirmed to be the most appropriate one for the present study. To save space, only the detailed model results from the random effect zero-inflated negative binomial model are presented hereafter.

Generally speaking, if the estimated coefficient of a parameter in a zero-crash state is positive, the probability in the zero state will increase and the predicted mean value of the crash count will decrease when the parameter increases. Meanwhile, if the estimated coefficient of a parameter in the negative binomial state is positive, then the predicted mean value of the crash count will increase. Therefore if the estimated coefficients of a parameter in the zero state and the negative binomial state are both positive or negative, it will be hard to tell whether the predicted mean value of the crash count will actually increase or decrease when the parameter increases. In this case, elasticity results will be important to provide more information. Elasticities are often computed to determine the marginal effects of the independent factors in panel data crash frequency models to provide some insight about the influence of different factors. The elasticities results are shown in Table 3 and some discussions are made by categories of parameters in the following.

### 4.1. Environmental Characteristics

The higher visibility is, the more likely the road segment will be in the zero-crash state. This implies that better visibility conditions decrease the crash frequency and bad visibility conditions increase the crash probability. Specifically, 1% decrease in visibility causes a 0.562% increase in the mean number of hourly crash frequencies, indicating that visibility is the most influential environment-related factors affecting crash frequencies on this I-25 corridor. Some other studies also highlighted the vital influence of real-time visibility condition on crash frequency [15,48,49,50].

The results in Table 2 suggest that crashes are more likely to occur with a lower crash frequency at night in the zero-crash state on I-25. It is noted that hourly traffic volume has also been included in the model which also decreases during night time. The results suggest that two different factors (*i.e.*, night and lower traffic volume) may jointly contribute to lower crash frequencies at night on I-25. Yet some studies found that nighttime increases the crash risk [51]. So more comprehensive studies may be needed in order to better disclose the nature of traffic safety at night when multiple contributing factors are involved.

The elasticity results in Table 3 suggest that crosswind speed slightly decreases crash frequency in the negative binomial state. It is known that driving under strong crosswind is pretty complex as it involves both vehicle performance and also driving behavior [52,53]. For the present study on I-25, it seems the benefits gained from more cautious driving likely outweigh the increased risk associated with vehicle performance under stronger crosswind. Usman *et al.* [48] found that higher wind speed is associated with higher number of crashes during winter storms. Because there are not many wind storms and complex terrain on I-25 in Colorado, it is found hard to draw a general conclusion about the influences on traffic safety from crosswind for all highways and a case-by-case study may still be needed.

Wet road surface is found to decrease crash frequency (negative coefficient in the negative binomial state as shown in Table 3). In contrast, chemically wet road surface contributes to the increase of crash frequency. Similar to crosswind, adverse road surface conditions (e.g., wet surface or chemically wet road surface) usually pose higher threats on vehicle stability, while at the same time, may alert the drivers to be more cautious on driving. Therefore, the final outcome of the impact from a particular variable depends on the cumulative safety effects from both the advantageous factors (e.g., more cautious driving behavior) and also the disadvantageous factors (e.g., slippery road surface with reduced friction coefficients). The influence on driving behavior from specific adverse environmental characteristics is very hard to be generalized only with the historical data used in this model. More studies on different highways with more extensive data are felt necessary in the future. In the meantime, the results in Table 3 show that chemically wet road surface is likely to be more critical than wet surface in terms of posing challenges on controlling the vehicle. The results also show that, given above discussed environmental variables included in the model, other hourly weather conditions like temperature and precipitation type, intensity and amounts have been found to not be significant in the models. Although the I-25 portion in this study has primarily flat terrain without experiencing frequent adverse weather common on highways with typical mountainous terrains, we still observe the significant effects from road surface and other environmental conditions in the crash frequency models. For those highways with typical mountainous terrain, the significance of refined-scale models considering detailed environmental and traffic conditions may become more substantial.

### 4.2. Traffic Characteristics

We use an instrumental indicator for speed limit and consider three options (<60, <65, <70 mph). Based on the best model fit, we choose a speed limit dummy indicator (1 if the legal speed limit is less than 60 mph, 0 otherwise) as the final input. In the negative binomial state, the indicator of low speed limit is found to increase crash frequencies (a positive coefficient). This finding is similar to those by Lee and Mannering [35], and instrumental indicator instead of the speed limit variable was also used in their study.

If actual average speed exceeds local speed limit, the Colorado DOT database will truncate it to speed limit of road segment. In the present study, the difference between speed limit and traffic speed instead of the absolute speed value is used; therefore the original real-time speed data from the Colorado DOT database do not exceed the local speed limit for each road segment. As a result, the difference between speed limit and traffic speed in this study has only nonnegative values, and it can reflect traffic congestion but not speeding behaviors. With regard to traffic speed, it is found that the larger difference between the legal speed limit and the traffic speed contributes to an increase of crash frequency (a positive elasticity coefficient in the negative binomial crash state). When the difference between speed limit and traffic speed is high, the traffic speed is usually low which indicates that congestion may occur. Therefore the model results show that the occurrence of congestion will increase the crash frequency on the study portion of I-25. Some existing studies also drew similar conclusion, and for example, Yu and Abdel-Aty [15] found that congested conditions in downstream traffic would contribute to an increase in the likelihood of multi-vehicle crashes.

Higher hourly traffic volume decreases the probability that the road segment would be in the zero-crash state (a negative coefficient). This indicates that higher hourly traffic volume may push the model to the negative binomial crash state, and then increase the crash frequency. Similar findings are also found in other studies [30,48]. Truck percentage is found to increase the crash frequency in the negative binomial crash state and also to increase the probability of road segments being in the zero-crash state. Therefore the trends of the elasticities of negative binomial state and zero-crash state are opposite. According to the elasticity results listed in Table 3 of both the negative binomial and the zero-crash states, higher truck percentage decreases the crash frequency. This finding can be found in some other studies [23,27]. One possible reason might be that as the percentage of trucks increases, other vehicle drivers will become more alert.

### 4.3. Temporal Characteristics

Turning to the estimation findings of temporal characteristics, we discover that a lower number of crashes are likely to occur during 4 am to 5 am, or sunrise period, within a day (negative coefficient in the negative binomial crash state). Within the whole year of 2010, a higher number of crashes are likely to occur during November. This could be due to unobserved effects associated with the early storm arriving Colorado and sudden temperature drop in November of 2010.

### 4.4. Road Characteristics

Several roadway geometric characteristics are found to significantly affect crash frequency along I-25 for both the non-zero and zero crash states. For the negative binomial state, crash frequency is found to decrease as the number of merging ramps per lane per mile increases. This phenomenon is likely related to the reduction in average speed of the traffic flow and/or the more cautious driving behavior with the number increase of merging ramps. Some studies found similar trends [54]. However, in some other studies, when the number of ramps (both merging and diverging ramps) per lane per mile increases, the crash probability increases as well [27,42]. Like some variables discussed previously, the findings indicate that the number of ramps may influence crash frequency in a more complex manner than people originally anticipated.

For I-25, the segment length of the highway is found to increase crash frequency in the negative binomial crash state and also to increase the probability of road segments being in the zero-crash state. The number of lanes is found significant with a positive coefficient in both negative binomial state and zero-crash state. If the number of lanes increases, crash frequencies decrease based on the elasticity results. The increase on the probability of zero-crash state is also possibly due to the relief of traffic congestion and more maneuvering space for vehicles to avoid being involved in a collision. The literature review shows that some studies [55] found similar results while some other studies found that crash frequency increases with an increase in the number of lanes due to more lane changing actions and in turn more conflicts [56,57].

On those segments with curvature, crash frequency is found to increase. The elasticity results show that 1% increase in degree of curvature is associated with a 0.385% increase of hourly crash frequency. While some studies found that a high degree of curvature is associated with an increase in crash likelihood [26], and more other studies found it to be positively associated with road safety [14,23,54,55]. Since curvature often works alongside other driving conditions (e.g., weather, slope, surface), it is not surprising to see the mixed effect of curvature on road safety from various studies.

The remaining service life for rutting index in the original CDOT database is used to define the rutting condition. The value of 100 indicates .15 inch or less rut. The value of 50 is the threshold that indicates no more remaining service life is left with an average rut depth of 0.55 inches. We choose a dummy variable named long remaining service life of rutting indicator (1 if the value of ruti is higher than 99, 0 otherwise) based on the best model fit (different thresholds of ruti have been tried). According to the elasticity results, the long remaining service life of a rut contributes to an increase of crash frequency (positive sign in negative binomial state). This implies that fewer crashes would occur when people likely tend to drive more slowly and cautiously on road segments with more ruts after sensing the rut-induced vibration and noise. Anastasopoulos and Mannering [27] found the effects of rut on crash frequencies vary significantly across roadway segments. Under excellent rutting condition, the majority of the road segments result in a decrease in crash occurrences, yet a few of the road segments still show the opposite. With regard to pavement conditions, good pavement condition indicator is found to decrease crash probability (negative sign in the negative binomial state). The definition of this indicator is that the condition of the road pavement for the primary direction is good. This phenomenon may reflect the improved vehicle performance due to better pavements.

## 5. Conclusions

The crash frequency model with refined scales in temporal domain is developed in this study. The major significance of this study is summarized in the following. Firstly, zero-inflated negative binomial model with site-specific random effect is developed to analyze the hourly crash frequency on highway segments with unbalanced panel data for the first time. Secondly, thanks to the high quality of the datasets, the present study can offer comprehensive coverage of various variables with refined scales, including environmental and traffic conditions, adding to the understanding of crash frequency modeling on major highways. Finally, the proposed refined-scale crash frequency models are developed with the monitoring data primarily from Road Weather Information System (RWIS), which is commonly available on many major highways around the country. As a result, similar technique can be applied to hundreds of major highways in the United States and other areas of the world without additional investments on data collection equipment.

Detailed data sets from I-25 in Colorado, including crash record, road design, real-time environmental and traffic conditions with refined temporal distributions, are adopted in the study. A number of critical factors about environmental characteristics, traffic characteristics, temporal characteristics and road characteristics are found significant to crash frequency. Some important findings are summarized in the following statements:

(1) Random effect zero-inflated negative binomial model is confirmed to be the most appropriate one according to the modeling fitness results. Elasticities are also computed to provide some important observations of the influence from different factors.
(2) The estimation results from the unbalanced panel data models show that both time-varying factors (e.g., visibility and hourly traffic volume) and site-varying factors (e.g., speed limit and number of lanes) may significantly influence the crash frequency on highways like I-25. Even for a typical highway without experiencing frequent adverse weather, the effects from road surface and weather conditions are found significant to the crash frequency model.
(3) Among all the significant variables, visibility condition is found to be the most influential environment-related factors affecting crash frequencies on I-25. Dark light condition (night), crosswind speed and wet road surface decrease crash frequency, while chemically wet road surface increases crash frequency. It is interesting that other hourly weather conditions, such as precipitation conditions and temperature, are not found to be significant on top of the current variables. It can be explained by the fact that precipitation and temperature does not influence crash likelihood directly, instead precipitation and temperature impact crash likelihood through changing visibility and road surface conditions. Since visibility and road surface conditions are already incorporated in the model, it is not surprising that precipitation and temperature becomes insignificant. Therefore the findings above underline the unique value and importance of the real-time road surface condition data to crash frequency studies.
(4) This paper reports the explorative effort on developing the new crash frequency models using detailed traffic, weather and road surface condition data in much more refined temporal scale (e.g., hourly data). Such a study bears a lot of potentials for engineering applications to make major highways safer and more resilient to adverse conditions.

## Figures and Tables

**Table 1:** Summary statistics of the data for observations.

Variable	Mean	Std. Dev	Minimum	Maximum
Crash frequency	0.004	0.066	0	4
**Environmental characteristics**				
Wet road surface	0.082	0.275	0	1
Chemically Wet road surface	0.037	0.188	0	1
Visibility (miles)	1.075	0.136	0.000	1.100
Cross wind speed (mph)	4.147	3.906	0.000	31.980
Average precipitation rate per minute(inches)	0.021	0.443	0	32
Average temperature (℉)	57.016	24.473	−1.333	159
**Traffic characteristics**				
Speed limit (mph)	61.027	5.327	55	75
Speed limit minus traffic speed (mph)	2.642	5.533	0.000	69.180
Hour traffic volume (in 1000 vehicles per hour)	2.916	2.101	0.030	14.988
Truck percentage (%)	6.215	1.922	2.800	10.700
**Temporal characteristics**				
Night	0.431	0.495	0	1
Sunset	0.062	0.241	0	1
November	0.095	0.294	0	1
4 am–5 am	0.040	0.196	0	1
**Road characteristics**				
Number of merging ramps per lane per mile	0.252	0.215	0.000	0.926
Segment length (miles)	1.014	0.769	0.236	4.500
Number of lanes	4.159	0.562	3	5
Remaining service life of rutting	97.013	2.984	86.000	100.000
Curvature (degree)	0.947	0.681	0.000	2.260
Good pavement condition	0.419	0.493	0	1
Median width (ft)	13.812	28.604	4	183
Outside shoulder width (ft)	10.335	2.181	6	15
Inside shoulder width (ft)	9.006	2.583	5	15
Grade (%)	−0.018	1.206	−2.334	2.334

**Table 2:** Random effect zero inflated negative binomial estimation results.

Variable	Estimate Coefficients	*t*-Statistic
**Zero-inflated State**		
Constant	−10.731	−5.84
*Environmental characteristics*		
Visibility (miles)	0.959	1.78
Wet road surface indicator (1 if the road surface is wet, 0 otherwise)	−1.663	−3.64
Chemically Wet road surface indicator (1 if the road surface is chemically wet, 0 otherwise)	−1.864	−5.04
*Traffic characteristics*		
Hourly traffic volume (in 1000 vehicles per hour)	−0.611	−9.06
Truck percentage (%)	0.439	5.95
*Temporal characteristics*		
Night indicator (1 if the time period is at night, 0 otherwise)	0.352	1.94
*Road characteristics*		
Segment length (miles)	0.755	4.60
Number of lanes	1.917	6.10
Good pavement condition indicator (1 if the pavement condition is good, 0 otherwise)	0.680	2.63
**Negative Binomial State**		
Constant	−10.673	−9.31
*Environmental characteristics*		
Cross wind speed (mph)	−0.013	−1.75
Wet road surface indicator (1 if the road surface is wet, 0 otherwise)	−0.529	−3.70
*Traffic characteristics*		
Low speed limit (1 if the speed limit is less than 60 mph, 0 otherwise)	0.387	1.83
Difference between speed limit and current traffic speed (speed limit minus traffic speed)	0.081	29.75
Truck percentage (%)	0.107	2.69
*Temporal characteristics*		
Sunset indicator (1 if the time period is during sunset, 0 otherwise)	−0.200	−1.88
November indicator (1 if the time period is in November, 0 otherwise)	0.292	3.20
4 am–5 am indicator (1 if the time period is between 4 am to 5 am, 0 otherwise)	−0.608	−1.96
*Road characteristics*		
Number of merging ramps per lane per mile	−1.072	−2.65
Segment length (miles)	0.786	5.44
Number of lanes	0.849	3.69
Curvature (degree)	0.406	3.07
Long Remaining service life of rutting indicator (1 if the value of ruti is higher than 99, 0 otherwise)	0.546	2.88
*α*	1.818	3.57
σ_i_ (site-specific)	0.484	7.54
Vuong statistic	4.48	
−2 Log Likelihood	15,145	
AIC (smaller is better)	15,197	
BIC (smaller is better)	15,250	

**Table 3:** Elasticity estimates for crash frequency (crash/hour).

Variable	Elasticity
*Environmental characteristics*	
Visibility (miles)	−0.562
Cross wind speed (mph)	−0.054
Wet road surface indicator (1 if the road surface is wet, 0 otherwise)	−0.243
Chemically Wet road surface indicator (1 if the road surface is chemically wet, 0 otherwise)	0.465
*Traffic characteristics*	
Low speed limit (1 if the speed limit is less than 60mph, 0 otherwise)	0.321
Difference between speed limit and traffic speed (speed limit minus current traffic speed)	0.215
Hourly traffic volume (in 1000 vehicles per hour)	0.637
Truck percentage (%)	−0.892
*Temporal characteristics*	
Night indicator (1 if the time period is at night, 0 otherwise)	−0.219
Sunset indicator (1 if the time period is during sunset, 0 otherwise)	−0.221
November indicator (1 if the time period is in November, 0 otherwise)	0.253
4am-5am indicator (1 if the time period is between 4 am to 5 am, 0 otherwise)	−0.836
*Road characteristics*	
Number of merging ramps per lane per mile	−0.270
Segment length (miles)	0.350
Number of lanes	−0.838
Curvature (degree)	0.385
Long remaining service life of rutting indicator (1 if the value of ruti is higher than 99, 0 otherwise)	0.421
Good pavement condition indicator (1 if the pavement condition is good, 0 otherwise)	−0.484

## Abbreviations

The following abbreviations are used in this manuscript:

ZINB	Zero-inflated Negative Binomial
RWIS	Road Weather Information System
ZIP	Zero-inflated Poisson
NB	Negative Binomial
CSP	Colorado State Patrol
CDOT	Colorado Department of Transportation
MM	Mile Marker
RCI	Roadway Characteristics Inventory
